# Nasal High-flow versus non-invasive ventilation in stable hypercapnic COPD: a preliminary report

**DOI:** 10.1186/s40248-015-0019-y

**Published:** 2015-09-03

**Authors:** Jens Bräunlich, Hans-Jürgen Seyfarth, Hubert Wirtz

**Affiliations:** Department of Respiratory Medicine, University of Leipzig, Liebigstr. 20, 04103 Leipzig, Germany

**Keywords:** COPD, Hypercapnia, Noninvasive ventilation, Nasal High-flow, Significant decrease in capillary pCO_2_, High-flow nasal cannula

## Abstract

**Background:**

There are no data available about effectiveness of Nasal High-flow (NHF)in chronic respiratory insufficiency.

**Methods:**

Eleven COPD patients with stable hypercapnia were adjusted to NHF-system with a flow of 20 l/min. After six weeks patients were switched to non-invasive ventilation (NIV) for another six weeks period.

**Results:**

NHF led to significant decreases in resting pCO_2_. Between the devices we found no differences in pCO_2_ levels.

**Conclusions:**

NHF may thus be an alternative treatment device in stable hypercapnic COPD patients.

## Background

Non-invasive ventilation (NIV) is a treatment option in patients with chronic respiratory insufficiency, hypercapnia and COPD [1]. A recent study shows a decrease in mortality by using NIV in patients with COPD [2]. In some of these patients, the tolerance of ventilation by mask is poor [3]. Nasal High-flow (NHF) represents a new method to support breathing. NHF devices are able to produce a heated and humidified airflow applied by large bore nasal prongs. Some investigations revealed benefits after extubation or cardiothoracic surgery in hypoxemic patient in comparison with Venturi mask or NIV [4, 5]. The study by Frat et al. documented a reduced intubation rate in severe hypoxemic patients. Surprisingly, the 90 days mortality rate might be better in NHF group in comparison with NIV and oxygen group [6]. However, these studies closed out patients with chronic respiratory insufficiency.

Several mechanisms could explain benefits of NHF. It´s assured that NHF generates a low positive airway pressure [7]. This could open atelectatic areas of the lung in acute hypoxemic failure e.g. pneumonia or prevent obstruction in chronic obstructive pulmonary disease. Also a wash-out effect in the upper airways may be another important mechanism [8]. Probably the dead space will be resolved because of placement of room air volume by NHF.

So, NHF exhibits various remarkable changes in breathing efforts in COPD patients: hypercapnia declines despite a decrease in respiratory rate and an increase in tidal volume [7]. This results in a reduced minute volume. The decline in hypercapnia suggests an improvement of alveolar ventilation. If this was verifiable NHF could be an alternative treatment option in patients with hypercapnia.

We initiated this clinical investigation in order to test this hypothesis and also to describe the possible long-time effectiveness of NHF in hypercapnic COPD patients.

## Methods

The study was approved by local ethics committee and patients gave their written informed consent (No. 123-2009-25052009; ClinicalTrials.gov Identifier: NCT02007772). Eleven COPD patients with a BMI below 30 kg/m^2^ and stable hypercapnia (≥ 50 mmHg) were included in the study in 2009–2011 (Table 1). Patients received blood gas analysis (BGA) for three times (further BGA also on exacerbation, screening and baseline visit). Last two investigations should exclude significant differences in capillary pCO_2_. Stable hypercapnia or disease was defined as an exacerbation-free time of six weeks. Hearth decompensation, acute illness or acute respiratory insufficiency were exclusion criteria. Four patients first screened during an exacerbation were included only if hypercapnia was still persistent following six weeks after the end of exacerbation. All other patients with hypercapnia in ambulatory BGA were referred to a pulmonologist. To prevent day-time variability in hypercapnia BGA was conducted at same day-time on every visit.

**Table 1:** Demographic data

Age	66.7 years
Gender m:f	7:4
FEV_1_	29.7 % pred.
FEV_1_%FVC	45.3 %
mean paCO_2_	53.7 mmHg
IPAP	16 cmH_2_O
EPAP	5.8 cmH_2_O

The primary outcome parameter was capillary pCO_2_ up to three hours following the end of treatment during the night. After an initial visit, patients were adjusted on NHF-system with a flow of 20 l/min with supplementary oxygen (TNI oxy, TNI medical AG, Würzburg, Germany). After 6 weeks patients were switched to NIV for another 6 weeks period (different systems). Patients were instructed to maintain stable oxygen supplementation that had to be stable during the 12 weeks of our study. Study visits included lung function and blood gas analysis. Statistics were done using Sigma Plot-software (Sigma Plot; Systat Software GmbH, Ekrath, Germany). Patients were instructed to use device more than 5 hours/ day. At the start of study only one device for home care use was available (maximum flow 20l/min). Only TNI device was able to provide accurate flow rates (measurements not shown).

## Results and discussion

**Fig. 1:**
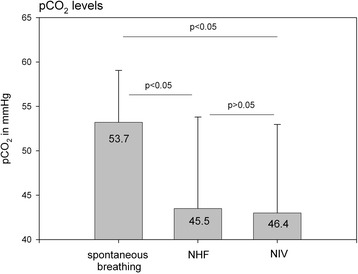
capillary pCO_2_-levels

Six weeks of NHF led to significant decreases in resting pCO_2_. After decreasing by NHF therapy, NIV was able to preserve normocapnia. No differences in pCO_2_ were observed between the two methods of non invasive ventilatory support (Fig. 1).

NHF so far has no clear defined areas of indications. Recently, published studies have described positive effects in acute hypoxemic failure [4–6]. Particularly with regard to mortality, we believe NHF therapy demonstrates significant benefits [6]. These studies recruited patients without any chronic respiratory diseases and hypercapnia. There are only few data available in such a patient cohort [7]. To our knowledge, this is the first description of long-time home care use in hypercapnic COPD patients.

Some authors focused on increased airway pressure, decreasing breathing rate and improvements in oxygenation. However, NHF effects appear more complex. As shown by several authors, an increase in airway pressure might be a helpful tool by supporting ventilation, but levels of achievable pressures are low [7]. Despite of this, significant effects on ventilation, with an increase in tidal volume, decrease in breathing frequency, and reduction in minute volume were observable [7]. As well shown in an animal study by Frizzola et al. a wash-out of the upper airways might be an important effect [8]. This point separates NHF from NIV via face mask. Since there was still a reduction in pCO_2_ NHF apparently increases the efficiency of breathing [7].

In this study we found a significant decrease in capillary pCO_2_ after using NHF for at least 5h/ day over 6 weeks. These results were similar to those in a following period using NIV. This allows to postulate two main findings. Firstly, the NHF decreases pCO_2_ despite of reduced minute ventilation. Further, it remains unclear what are the main effects of working, but the reduction in hypercapnia demonstrates that NHF is able to affect alveolar ventilation. By using NIV normocapnia was stabilized. One could speculate that NIV (with low pressure levels) is able to decrease capillary pCO_2_ in the same way.

There are several limitations to our study. First, patients were not randomised and the study was monocentric. All participants started with NHF. The initial hypercapnia was moderate. Patients may have more difficulty tolerating high NIV peak airway pressure levels. Because of small sample size, a beta-error might be possibly. We only used a low flow with 20 l/min and the reason was lack of availability of home care devices with constant flow.

## Conclusions

NHF may thus be an alternative treatment device in stable hypercapnic COPD patients. A multicentric and randomised investigation is now in planning and will be conducted in order to verify the findings of this observational investigation.
